# Reconstruction of a Self-sustaining System of Oscillating Glycolytic Reactions in Artificial Microreactors

**DOI:** 10.32607/actanaturae.27965

**Published:** 2026

**Authors:** O. M. Alekhina, P. V. Bashkirov, A. S. Silantyev, S. P. Dudik, D. S. Matyushkina, O. V. Shcherbakova, V. M. Govorun

**Affiliations:** Simple Systems Laboratory, Research Institute for Systems Biology and Medicine, Moscow, 117246 Russia; Microfluidics and Nanofluidics Laboratory, Research Institute for Systems Biology and Medicine, Moscow, 117246 Russia; Laboratory of Molecular Pathophysiology, Lopukhin Federal Research and Clinical Center of Physical–Chemical Medicine, Federal Medical-Biological Agency, Moscow, 119435 Russia

**Keywords:** glycolysis, oscillations, cell-free systems, microcompartments, microfluidics, *in vitro* transcription

## Abstract

Synthetic cell design is one of the daunting challenges of modern biology. While the genome synthesis problem has already been solved technologically, the understanding of the molecular composition of the intracellular environment required for initiating artificial life still remains elusive. One of the mechanisms of self-organization in prebiotic systems is the oscillatory processes (oscillations) arising from feedback regulation. Such dynamics are inherent to glycolysis as the metabolic core of the cell. The objective of this study was to reconstruct the oscillatory glycolytic chain and investigate its behavior upon confinement to microcompartments and upon interaction with nucleic acids. We used the *Saccharomyces cerevisiae* extract as a model and demonstrated the presence of fluctuations in the concentrations of glycolytic metabolites: ATP, NADH, hexose-6-phosphates, glyceraldehyde-3-phosphate, 3-phosphoglycerate, and phosphoenolpyruvate (PEP). Moreover, the PEP and pyruvate concentrations had a dramatic effect on the stability of oscillations. We also demonstrated that the reduction of the system volume accompanying the encapsulation into a microchip leads to a more rapid onset of oscillations. Furthermore, synthetic mRNA and DNA were found to reduce the amplitude and period of oscillations in a translation-independent manner. Hence, this study demonstrates the feasibility of reconstructing metabolic oscillations in microvolumes and reveals the effect of nucleic acids on the glycolytic dynamics, which is important for the development of protocellular systems.

## INTRODUCTION

The concept of life defined as a system based on the encoding, storage, and expression of genetic information by biopolymers – nucleic acids (NAs) and proteins – is a key principle of modern biology. However, in the context of the origin and early evolution of life, there arises a pivotal question as to whether a complex, self-organizing, and self-sustaining biochemical system could have existed before or independently of the emergence of these high-molecular-weight structures. In theory, primary prebiotic systems could have been metabolic networks consisting of low-molecular-weight compounds, with their stability and reproducibility ensured by mechanisms such as autocatalysis, feedback, and the emergence of spatiotemporal dissipative structures [1, 2], including biochemical oscillations [3]. The evolutionary scenario of primary metabolism assumes that primitive protocells could have maintained homeostasis and adaptation through the dynamics of metabolic fluxes long before the intricate replication and translation machinery evolved [4, 5].

Oscillations, observed at all levels of the organization of living matter [6], are an archaic and universal regulatory principle that is capable of synchronizing heterogeneous biochemical processes and eliciting a coordinated response to environmental changes. Oscillations are caused by the nonlinear dynamics of enzymatic networks and embody the fundamental principles of biological self-organization. Glycolysis, the central and evolutionarily ancient pathway of glucose catabolism, is the key model for studying these pre-coding regulatory mechanisms [7]. Back in the late 1950s and early 1960s, several research teams observed non-steady-state changes in oxygen uptake and NADH fluorescence in cells [8, 9]. However, it was B. Chance et al. (1964) who systematized early observations and unequivocally showed that glycolysis was a source of oscillations [10, 11] arising within a complex enzymatic network through nonlinear dynamics and allosteric regulation, primarily of the phosphofructokinase (PFK) enzyme [12].

The modern studies focusing on glycolytic oscillations have substantially expanded the original concepts, as they proceeded from investigating oscillations in cell suspensions to analyzing their complex spatiotemporal dynamics. The advances in microfluidic technologies have made it possible to study glycolysis under conditions similar to those of the dimensions of a real cell, where the significant role of diffusion limitations and stochastic fluctuations starts to lend itself to observation [13, 14]. In turn, the encapsulation of glycolytic systems into liposomes or coacervate droplets paves the way towards the design of synthetic oscillators and modeling the behavior of protocells [15].

Nonetheless, although considerable progress has been made, many aspects of glycolytic oscillations remain vaguely understood. In particular, the potential role of NAs in the dynamics of oscillatory processes is still an open question. NAs have conventionally been regarded solely as carriers of genetic information. But is there a direct impact of polynucleotides on metabolic oscillations that is not mediated by protein synthesis? Could NAs have fulfilled both an informational and regulatory function, modulating the activity of primitive metabolic networks during the early evolution stages? The answers to these questions can possibly shed light on the potential pathways of coevolution of metabolism and information systems.

This study focused on the conditions required for the emergence and key parameters of glycolytic oscillations in cell-free extracts from the yeast *S. cerevisiae*. The critical metabolic factors needed for initiating and sustaining the oscillatory regime were identified, and the dynamics of the intermediates were analyzed. Moreover, the role played by diffusion processes and spatial organization in the oscillation kinetics upon physical confinement (encapsulation into a microvolume) was assessed, which is important for evaluating the intracellular occurrence of the observed reactions. Furthermore, for the first time, we experimentally tested the hypothesis that synthetic NAs have a direct, translation-independent impact on the parameters of glycolytic oscillations.

## EXPERIMENTAL

**Producing yeast extract**

*S. cerevisiae* cells (DBY-α strain) from a frozen stock were plated on YPD agar medium (20 g/L peptone, 20 g/L glucose, 10 g/L yeast extract, 15 g/L agar) and cultured at 30°C for 12 h. A single colony was inoculated into 80 mL of “nutrient-limited” YNB medium (6.7 g/L YNB without amino acids (Sigma, USA), 3 g/L yeast extract, 10 g/L glucose, 100 mg/L ampicillin) and cultured at 30°C for 12 h under vigorous stirring. The resulting culture was used to inoculate eight 2 L flasks, each containing 750 mL of YNB medium with 100 mg/L ampicillin (1% inoculum), and cultured until glucose was completely exhausted (6–8 h). The glucose levels were monitored using glucose test strips. Cells were pelleted by centrifugation (6,000 rpm, 4°C, 25 min), washed twice with 0.1 M potassium phosphate buffer (pH 6.8), and resuspended in 150 mL of the same buffer. After incubation at 25°C and 170 rpm for 3 h (the starvation stage), the cells were collected by centrifugation (5,500 rpm, 4°C, 15 min) and resuspended in a minimal volume of 0.1 M potassium phosphate buffer (pH 8.0; final suspension volume ≤ 30 mL). The cells were disrupted employing a French press (three passages, 1000 atm); the lysate was centrifuged at 15,000 *g* (4°C) for 30 min; the supernatant was additionally centrifuged (1.5 h, 100,000 *g*, 4°C) in a swinging-bucket rotor. Aliquots of the extract were frozen in liquid nitrogen and stored at −80°C. Protein concentration was determined using the Bradford assay.

**Detection of NADH oscillations**

An aliquot of the extract was thawed on ice, and 100 mM trehalose from a stock solution (1.6 M) was added. When needed, metabolites (glucose-6-phosphate, fructose-6-phosphate, fructose-1,6-bisphosphate, NAD, NADH, ATP, ADP, AMP, pyruvate, and PEP) were added at physiological concentrations (1–5 mM) from 50–100 mM aqueous stock solutions. The extract was diluted with 0.1 M potassium phosphate buffer (pH 6.8). The reaction mixture (150 µL) was transferred to a quartz cuvette (5 mm) and placed into a thermostated cell (25°C) in a Hitachi F-2500 spectrofluorometer. NADH fluorescence was measured every 10 s at λ_em_ = 455 nm (λ_ex_ = 355 nm) for several hours. When metabolites needed to be added, the cuvette was removed, the metabolite was quickly added, stirred, and returned to the setup.

**Detection of ATP oscillations**

A luciferase-based assay was employed to simultaneously measure the ATP consumption and synthesis levels during glycolytic oscillations. Luciferin (0.15 mM) and luciferase (4 µg/mL; Sigma) were added to the extract. Luminescence was detected on a Tecan Infinite MPlex multiplate reader (Greiner 384 flat-bottom white polystyrene plates) at λ_em_ = 500–600 nm for several hours with automated shaking.

**HPLC-MS**

Reaction mixture samples (25 µL) were collected as quickly as possible and immediately mixed with 200 µL of ice-cold methanol (−80°C), vortexed vigorously, and incubated at −80 °C for at least 15 min. The samples were thawed on ice for 5–10 min and centrifuged (30 min, 15,000 rpm, 4°C). The supernatants were transferred to new tubes, and 1 µL of internal standard (1 mg/mL glutamate ^13^C_5_) was added. The contents of the tubes were dried in a SpeedVac vacuum concentrator at 30°C and stored at −80°C.

An analysis was performed using a SCIEX 4500 QTRAP tandem mass spectrometer coupled to a Shimadzu 30ACMP liquid chromatography system. The source settings were as follows: curtain gas (CUR) pressure, 24.1 kPa; source gas 1 (GS1) pressure, 37.9 kPa; source gas 2 (GS2) pressure, 37.9 kPa; heated gas temperature (TEM), 450°C; and ionization needle voltage (IS), −4500 V. Samples were supplied using an electrospray ionization source. The analytes were detected in the multiple reaction monitoring mode (MRM) with negative ionization: PEP 167→79 (63); hexose-6-phosphate 259→97 (79); glyceraldehyde-3-phosphate 169→97 (79, 151); glucose 239→179 (89, 59); 3-phosphoglycerate 185→97 (79); and NADH 665.4→524.1 (79).

The components of the analyzed sample were separated by hydrophilic interaction liquid chromatography on a Phenomenex Kinetex HILIC column (2.6 µm, 50 × 2.1 mm): phase A (100% H_2_O; 10 mM ammonium acetate; 1 µg/mL citric acid; pH 7.0); phase B (10% H_2_O; 90% acetonitrile; 10 mM ammonium acetate; 1 µg/mL citric acid; pH 7.0). The flow rate was 0.45 mL/min, the injection volume was 1 µL; the temperature of the column thermostat was 45°C. Chromatographic separation of the analytes was performed in the gradient mode: 0 min 100% B; 0.5 min 100% B; 4.5 min 90% B; 9 min 50% B; 11 min 50% B; 11.1 min 100% B; 13 min 100% B.

*Tables 1* and *2* list the parameters of calibration curves of the MRM transitions for quantifying the target compounds and their intraday reproducibility, respectively. The calibration curves for the analytes were plotted using linear regression with the normalized 1/x^2^ function. A correlation coefficient ≥ 0.9 was considered acceptable. Each calibration for the compounds studied comprised five points in duplicate with a 2.5-fold dilution step.

**Table 1:** Parameters of the calibration curves of the MRM transitions for quantification of the target compounds

Compound	LLOQ^1^, ng/mL	ULOQ^2^, ng/mL	R²	Slope, o.u./ng mL^-^¹	Intercept term, o.u.
PEP	101	3,960	0.9937	1,392.2	21,131
Hexose-6-P	46	1,800	0.9895	17.6	-117.8
Glyceraldehyde-3-P	46	1,800	0.9986	1,103.3	75,300
Glucose	46	1,800	0.9937	3,556.9	-25,061
3-Phosphoglycerate	50.7	1,960	0.9942	75.15	-3,735.6
NADH	25.6	1,000	0.9364	13.89	309.7

^1^LLOQ – lower limit of quantification. ^2^ULOQ – upper limit of quantification.

**Table 2:** Intraday reproducibility for the analytes

Compound	Area, %	Normalized area, %
PEP	11.50	15.20
Hexose-6-P	3.80	9.10
Glyceraldehyde-3-P	4.50	7.40
Glucose	5.80	8.0
3-Phosphoglycerate	10.60	14.10
NADH	8.10	9.10

**Inverted micelle synthesis**

The lipid POPC (palmitoyl-oleoyl-phosphatidylcholine) was dried from chloroform and dissolved in mineral oil (Sigma, lightoil) to a concentration of 2 mg/mL. In order to form micelles, 120 µL of the oil phase was mixed with 2 µL of the inner solution (60 : 1 ratio) and emulsified by vigorous shaking for 1 min. The resulting emulsion was applied onto a coverslip. NADH was visualized by confocal fluorescence microscopy using a UV lamp as a light source and an optical filter (λ_em_ = 440–490 nm; λ_ex_ = 325–375 nm).

**Encapsulation into a microfluidic chip**

The master mold was fabricated by photolithography and plasma etching of a silicon wafer. The wafer surface was made hydrophobic using hexamethyldisilazane vapor (150°C, 40 min), followed by application of the S1813 photoresist via spin coating (3,000 rpm, 2 min). Exposure was performed using a photomask and a 350 W mercury lamp. UPF-1B developing solution (Frast, Russia) was used for development. Silicon was etched on a Plasmalab 100 system (Oxford Instruments, UK) using the three-stage Bosch process. The residual photoresist was removed with acetone, followed by rinsing with deionized water.

In order to fabricate the microfluidic chip, the master mold was filled with polydimethylsiloxane (PDMS) and polymerized at 80°C for 2 h. The cured PDMS was separated from the mold; chips were cut out, and inlet orifices were punched. After oxygen plasma treatment, the chip was irreversibly attached to a glass slide. The chips contained a system of multiple cavities (20–100 µm in diameter) connected to a common channel. The cavities were filled with concentrated yeast extract containing 100 mM trehalose. The chip was mounted on the sample stage of a fluorescence microscope, and NADH fluorescence signal in all the cavities was continuously recorded.

***In vitro* transcription**

Transcription from linearized plasmids was carried out using T7 RNA polymerase employing the Gurevich technique [16]. The reaction mixture contained 0.2 M HEPES-KOH (pH 7.5), 6 mM of each ribonucleoside triphosphate, 0.1 mg/mL BSA, 2 mM spermidine, 40 mM DTT, 28 mM MgCl_2_, 0.2 U/µL RNase inhibitor (Thermo Fisher, USA), 3 U/µL T7 RNA polymerase (ThermoFisher), and 0.05 mg/mL linearized DNA template. The reaction was conducted at 38°C for 2 h and stopped by adding an equal volume of phenol/NaOAc (pH 5.0), followed by stirring and centrifugation (3 min at 20,000 *g*). The upper phase was collected, while the lower phase was re-extracted with an equal volume of water. The pooled aqueous phase was treated with 1/2 volume of chloroform and centrifuged. The upper phase was transferred to a new tube, and LiCl was added until a concentration of 3 M. After 40-min incubation on ice, RNA was precipitated by centrifugation (20,000 *g*, 15 min). The pellet was dissolved in 100 µL of water; 0.9 volumes of 4 M ammonium acetate and 2.5 volumes of ethanol were added. After 1-h incubation at −80°C, RNA was precipitated, dissolved in 100 µL of water, and then 0.1 volumes of 4 M sodium acetate (pH 5.0) and 2.5 volumes of ethanol were added. The mixture was incubated at −80°C for 1 h and precipitated. The pellet was washed twice with 80% ethanol, dried, and dissolved in deionized water. RNA concentration was determined using a NanoDrop spectrophotometer (λ = 260 nm). The product yield was ~40 µg of RNA per µg of DNA template.

## RESULTS

**Initial enzyme and substrate concentrations determine the occurrence and kinetic characteristics of glycolytic metabolite oscillations in *S. cerevisiae* cell extracts**

The ability of the yeast extract to undergo glycolytic oscillations depends on the initial concentrations of enzymes and substrates. Three conditions need to be met: (1) yeast cultivation in the glucose-limited medium until complete glucose exhaustion (switching to consumption of ethanol produced earlier); (2) incubation of cells in a buffer (the starvation stage) to equilibrate the metabolite pools; and (3) continuous supply of the glycolytic substrate at a constant rate [17]. In our system, 100 mM trehalose was used as a source of glucose. The oscillations were detected by monitoring NADH fluorescence.

All the experiments were conducted for at least three independent series of the extract. Since the yeast cell culture cannot be completely synchronized [18] because of differences in the ratios between the key intracellular metabolite concentrations at the time of cell disruption, we observed variations in the kinetic parameters of oscillations. The effects of perturbations introduced to the system and the observed patterns were qualitatively reproducible between the extract series. All statistical parameters were calculated separately for experiments performed with each extract series.

At a protein concentration of 102 mg/mL, the extract with trehalose started to oscillate after 59 ± 1 min (*n* = 3); the oscillations continued for at least 3 h (*Fig. 1A*). The oscillation period was 7–9 min; the amplitude was ~12% of the total fluorescence signal and gradually decreased over time. Extract dilution increased the duration of the lag phase: at a protein concentration of 54 and 15 mg/mL, the oscillations started after 98 ± 1 min (*n* = 3) and after 127 ± 2 min (*n* = 3), respectively (*Fig. 1B, C*). The oscillation duration decreased, but their period and maximum amplitude increased. In the most diluted extract (15 mg/mL), oscillations emerged only after 0.5 mM NADH and 3 mM ATP had been added, as their endogenous concentrations were insufficient to induce oscillations. Unless otherwise specified, further experiments were performed using extracts of identical total protein concentrations (72 mg/mL). The high protein concentrations may cause quenching of the fluorescence signal, so we compared changes in the NADH fluorescence signal qualitatively rather than quantitatively.

**Fig. 1:**
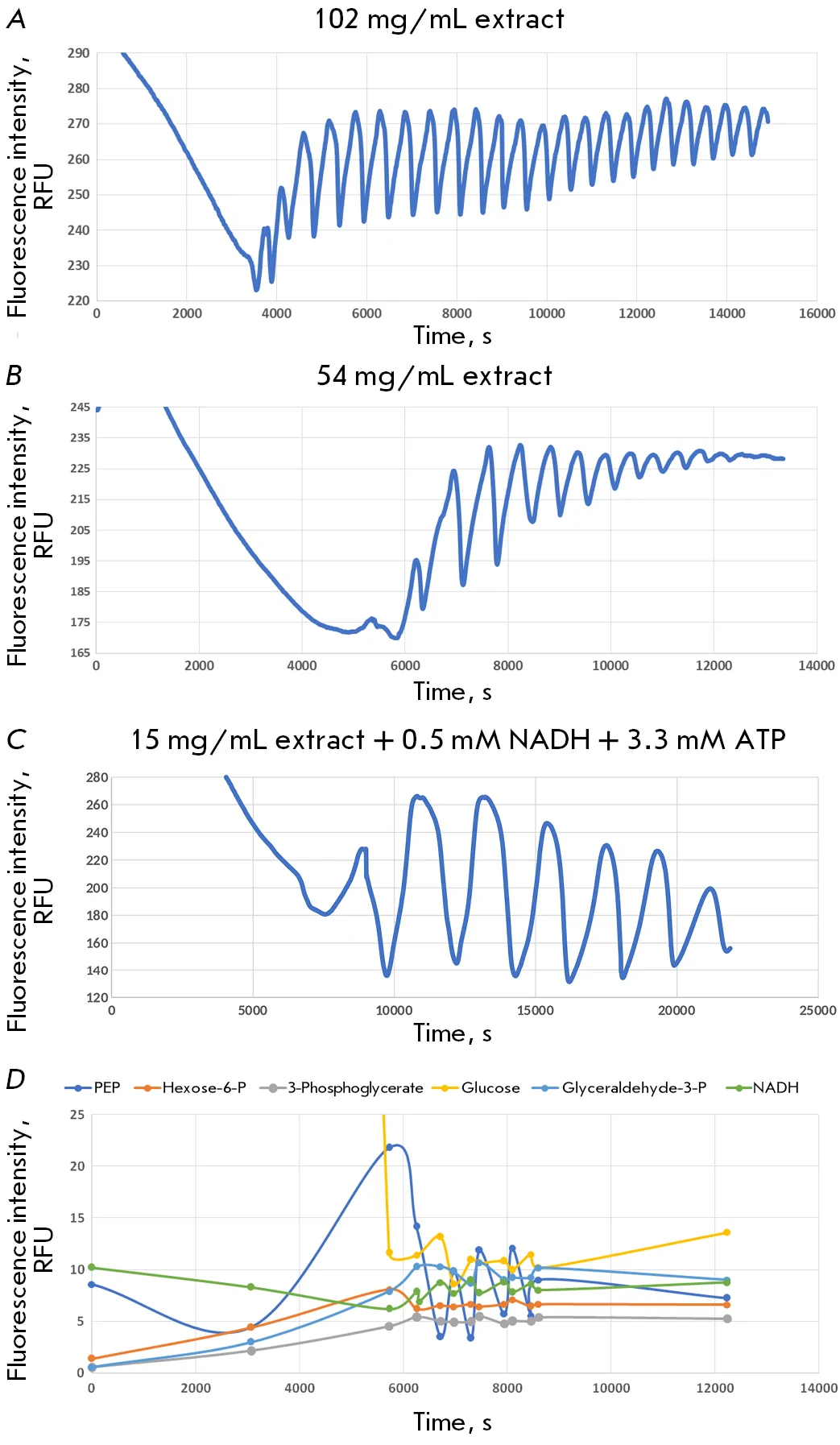
Oscillations of glycolytic intermediates in yeast extract. (*A*, *B*, *C*) – representative (for at least three replicates) time dependence curves of NADH fluorescence intensity at different protein concentrations in yeast extract: (*A*) 102 mg/mL; (*B*) 54 mg/mL; (*C*) 15 mg/mL, 0.5 mM NADH and 3 mM ATP added. (*D*) The result of identification and quantitative assessment of glycolysis intermediates by HPLC/MS using internal isotope standards (a representative diagram of three replicates)

We conducted HPLC-MS with isotope-labeled standards to confirm that periodic changes were observed not only for NADH but for glucose, hexose-6-phosphates, glyceraldehyde-3-phosphate, 3-phosphoglycerate, and PEP as well (*Fig. 1D*). Intermediate concentrations change substantially during the lag phase to attain the threshold values required for transition to the self-oscillating regime. Once self-sustained oscillations are established, each metabolite is maintained within a characteristic narrow concentration range.

**The ATP-to-NADH ratio in the yeast extract plays a crucial role in the oscillation dynamics**

The addition of individual metabolites before the incubation revealed variations in their effects on the oscillation kinetics. NAD^+^ and NADH did not affect the onset time of oscillations but shortened their duration (*Fig. 2A*). Hexose phosphates slightly accelerated the onset of the oscillatory phase (*Fig. 2B*). By contrast, the addition of ATP, pyruvate, or PEP induced oscillations almost immediately, although initially they were irregular and had a longer period (*Fig. 2C*). Since pyruvate and PEP are involved in the reactions coupled to ATP synthesis, we put forward a hypothesis that ATP deficiency in the initial extract is a factor responsible for the long lag phase, since ATP is required for the first step of glycolysis.

**Fig. 2:**
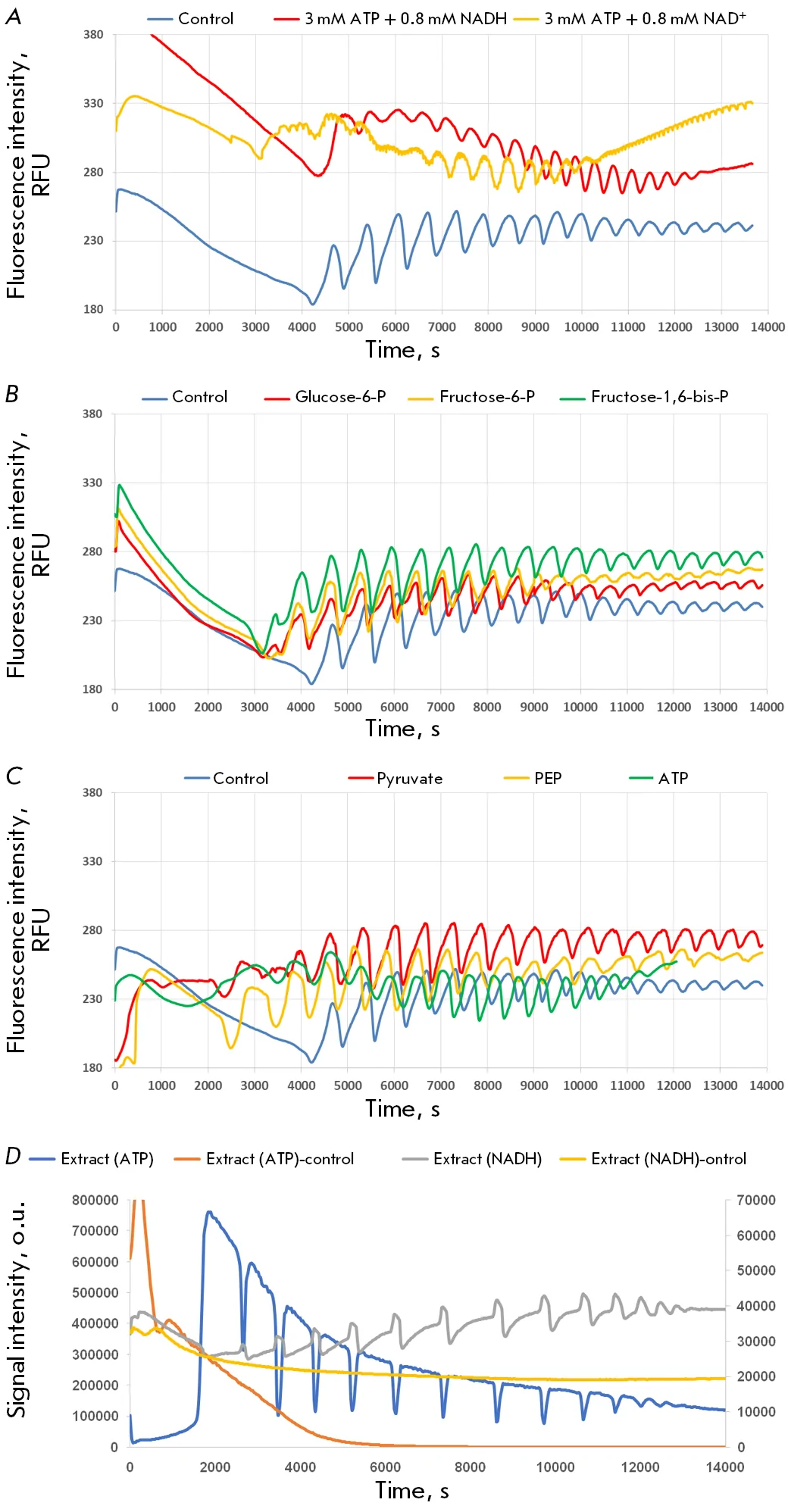
The effect of glycolysis metabolites on oscillations of NADH concentration in yeast extract when they are added at the beginning of the reaction. (*A*, *B*, *C*) representative (for at least three replicates) time dependence curves of NADH fluorescence intensity in the presence of additional amounts of glycolytic metabolites in the yeast extract: (*A*) 3 mM ATP with 0.8 mM NAD^+^ or 0.8 mM NADH; (*B*) 3 mM fructose-1,6-bisphpsphate, or fructose-6-phpsphate, or glucoso-6-phosphate; (*C*) 3 mM pyruvate, or PEP, or ATP. (*D*) Oscillations of NADH (fluorescence) and ATP (luciferin luminescence) upon simultaneous detection in the yeast extract in the presence or absence of 100 mM trehalose (representative curves of three replicates)

Due to the compatibility of the luciferase system with the yeast extract, we, for the first time, simultaneously performed real-time detection of the ATP and NADH dynamics (*Fig. 2D*). NADH was found to display complex bimodal dynamics, whereas ATP oscillated in antiphase with NADH: the abrupt transient drops in the ATP level were accompanied by burst-like increases in the NADH level. These findings agree with the model in which ATP inhibits the key glycolytic enzymes (PFK and PK), and its accumulation slows down the pathway, thus initiating a new cycle. Our data convincingly demonstrate that a certain level of the two cofactors, ATP and NADH, is absolutely necessary for oscillatory dynamics in glycolytic reactions to be induced.

**Pyruvate and PEP added to the yeast extract induce perturbations in glycolytic oscillations**

In order to identify the metabolites critical for sustaining oscillations, we added them at a concentration of 2 mM during the self-sustaining oscillation phase. ATP, ADP, AMP, NAD^+^, and fructose-1,6-bisphosphate had virtually no effect on the oscillation dynamics (*Fig. 3B,C*). By contrast, the addition of pyruvate or PEP abruptly reduced the NADH fluorescence and halted the oscillations; the oscillations resumed after ~ 30 min in most cases (*Fig. 3A*). PEP had a more drastic effect, especially when added at the NADH minimum, where the concentration of endogenous PEP was maximal (*Fig. 3D*). Extra PEP added during this phase apparently drives the system out of the self-sustaining state, causing oscillation relaxation.

**Fig. 3:**
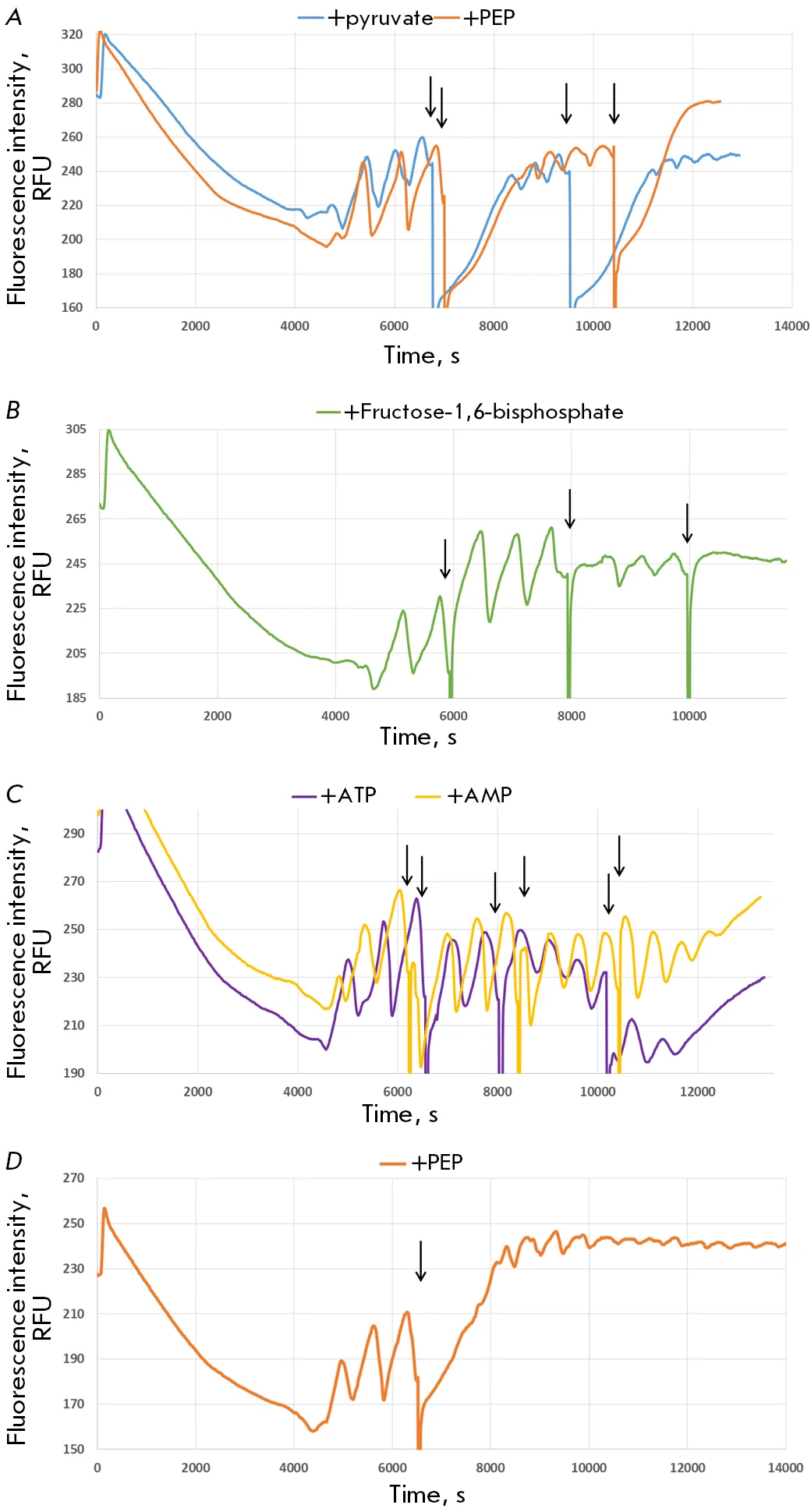
The effect of glycolysis metabolites on oscillations of NADH concentration in the yeast extract when adding them to sustained oscillations. (*A*, *B*, *C*) The representative (for at least three replicates) time dependence curves of NADH fluorescence intensity when adding amounts of 0.5–2 mM glycolytic metabolites in the yeast extract at the time points indicated with arrows: (*A*) pyruvate or PEP; (*B*) fructose-1,6-bisphpsphate; (*C*) AMP and ATP. (*D*) PEP added at the minimal NADH concentration

**Reconstruction of the oscillating yeast system in microvolumes**

The encapsulation of the concentrated extract (108 mg/mL) with trehalose into inverted micelles demonstrated that the oscillation pattern was dependent on the compartment size (*Fig. 4A*). The oscillations in droplets 1–2 mm in diameter were similar to those in bulk (the oscillation period was ~ 12 min). No oscillations were detected in micron-sized droplets. We hypothesized that the reason behind it could be the accumulation of CO_2_ formed upon pyruvate decarboxylation. In closed microvolumes, the partial pressure of CO_2_ rose rapidly, leading to oscillation inhibition.

**Fig. 4:**
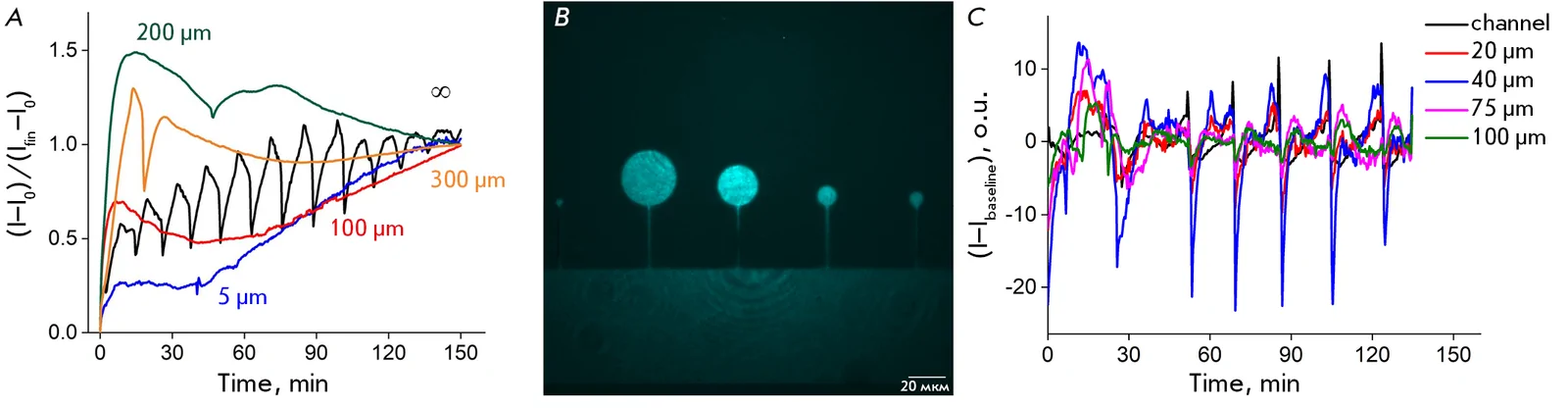
The kinetic parameters of oscillations inside artificial microcompartments. (*A*) Time dependence curves of NADH fluorescence intensity in the yeast extract, enclosed in inverted micelles of different diameters; (*B*, *C*) the result of NADH fluorescence measurement in yeast extract introduced into the cavities (20–100 μm in diameter) of a PDMS microchip by confocal microscopy. (*B*) The view of the microchip with the injected extract; and (*C*) time dependence curves of NADH fluorescence intensity for different cavity sizes

We used microfluidic chips composed of PDMS, a material capable of absorbing gases, to overcome this effect. The chips had cavities 20–100 µm in diameter (*Fig. 4B*). After the chips had been filled with the trehalose-containing extract, the fluorescence microscopy measurements of NADH fluorescence revealed that oscillations took place in all the cavities regardless of their size (*Fig. 4C*). It is important to mention that when approaching the oscillatory regime, the fluctuations in fluorescence intensity within cavities of different diameters synchronized with oscillations in the common channel feeding them (within the measurement frequency of ~ 20 s). These findings confirm that CO_2_ needs to be removed in order to maintain self-sustaining oscillations of the glycolytic reaction in the microvolume.

**The effect of nucleic acids on oscillations**

Since NAs are not spatially separated from metabolic processes in living systems, we decided to focus on the effect of DNA and RNA at different levels of structural organization on the kinetic parameters of the glycolytic oscillating system. We took a plasmid carrying the luciferase gene from the firefly *Photinus pyralis* controlled by a high-efficiency T7 phage RNA polymerase promoter. RNA (1,882 nucleotides) was obtained from this plasmid using *in vitro* transcription. According to the predictions, this RNA had well-defined secondary and tertiary structures. The mRNA obtained at the maximum possible concentration (16 µM for luciferase mRNA and 8 µM for hatA mRNA) was added to the yeast extract, together with trehalose, and inverted micelles were produced. In all the cases, the addition of RNA changed the kinetic parameters of oscillation (*p* < 0.05) by reducing the oscillation period. The frequency of oscillations increased by 18 ± 16% (SD, *N* = 9) in the presence of RNA. This effect was independent of the specific nucleotide sequence of RNA (*Fig. 5B–D*). Moreover, a similar change in oscillation frequency by 17 ± 13% (SD, *N* = 3) was also observed after adding DNA (*Fig. 5D*). The control experiments showed that the effect of RNA/DNA was not attributed to their ability to bind magnesium ions, since an increase in magnesium concentration did not significantly change the characteristic frequency of glycolytic oscillations (*Fig. 5A,B*).

**Fig. 5:**
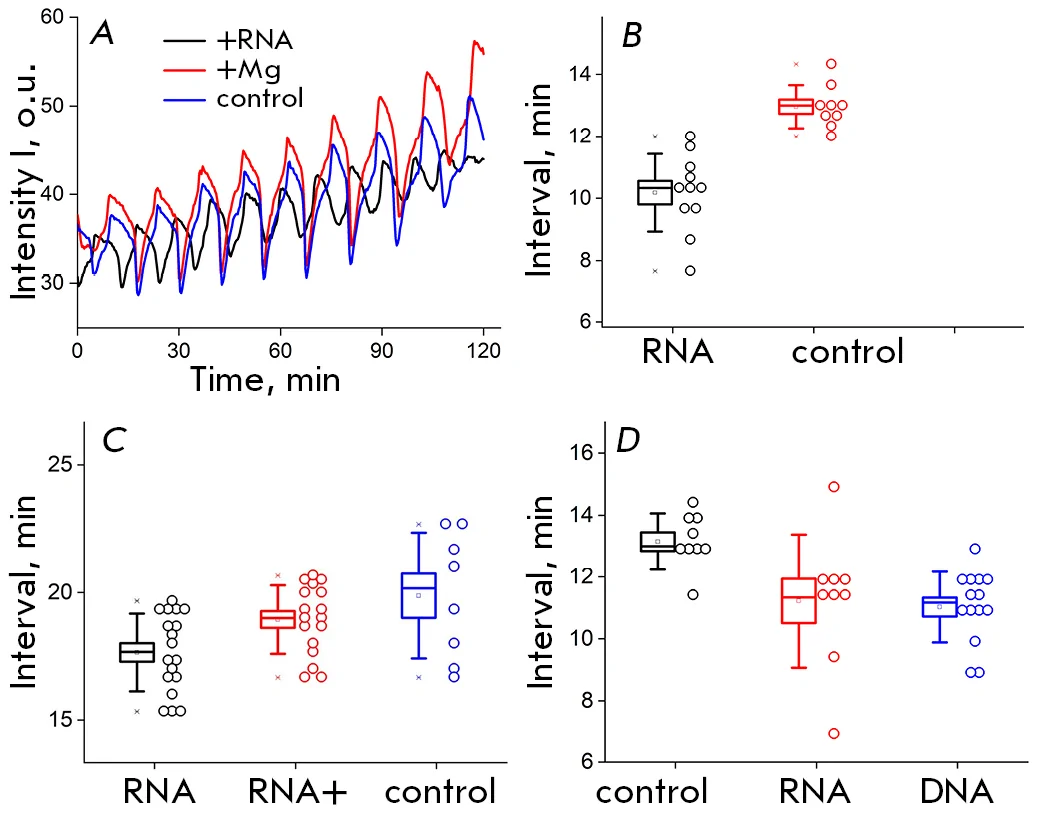
Effect of nucleic acids on the dynamics of NADH oscillations in the yeast extract encapsulated into inverted micelles, detected according to fluorescence by confocal microscopy. (*A*) Fluorescence intensity versus time; (*B*, *C*, *D*) duration of the oscillation period (s) in time in the presence of: (*B*) 16 μM luciferase mRNA; (*C*) 8 μM *hatA* mRNA in the presence or in the absence of thiamine (20 μM); and (*D*) luciferase mRNA and DNA of a linearized plasmid carrying the luciferase gene

Next, we tested whether the effect of RNA on oscillations is related to its well-defined spatial structure. For this purpose, we constructed a plasmid in which the DNA region carrying the *hatA* gene of *Mycoplasma gallisepticum* was placed under the control of the T7 RNA polymerase promoter. A unique feature of this gene is that its expression can be regulated at the translational level using a riboswitch.

A short 457-nt RNA synthesized using this construct carried a riboswitch sequence in its 5′-untranslated region. Upon interaction with thiamine, it induced folding of the nucleotide chain into a structured translation-inaccessible conformation.

The experiments where this mRNA was added to the yeast extract containing trehalose in the presence and in the absence of thiamine showed that both poorly and well-structured polyribonucleotides affect the kinetic parameters of glycolytic oscillations (*Fig. 5C*). Therefore, it can be inferred that RNA is capable of altering the intermediate flux rates in metabolic circuits and that this effect is weakly dependent on the primary and spatial RNA structure.

We also studied the effect of DNA on glycolytic oscillations in the yeast extract, although DNA is usually isolated from metabolic circuits in eukaryotic cells. In these experiments, we used plasmids carrying different nucleotide sequences, which were linearized using restriction enzymes and then used in the same amounts as RNA (calculated on the basis of nucleotide content: 8 and 4 µM, respectively). We found that, regardless of its sequence, DNA reduced the oscillation period, like RNA did (*Fig. 5D*).

Since the effect of NAs turned out to be nonspecific, we assumed that it could be related to the molecular charge. In order to test this hypothesis, we added dextran sulfate in an amount so that its charge was equivalent to that for NAs used earlier. At the begining of oscillation recording, dextran sulfate had no effect on oscillation parameters but caused precipitation of the components over time. Therefore, the use of polyelectrolytes to control the amount of negatively charged functional groups is rather challenging in multicomponent systems such as yeast extracts.

## DISCUSSION

The central question of this study is whether complex metabolic systems can have stable self-organization and be autonomously regulated in the absence of intricate biopolymeric (DNA, RNA, and protein) regulatory networks. In the prevailing paradigm, the primary role in the function of the modern cell is assigned to the system encoding and implementing genetic information. However, the emergence of long biopolymers during the early evolution stages must have been preceded by the formation of self-sustaining protometabolic networks based on low-molecular-weight catalysts and autocatalytic cycles [19, 20]. These systems could have formed under conditions far from equilibrium, and their stability was apparently ensured by mechanisms resembling dissipative structures and chemical oscillators [2]. Our findings obtained using a model of glycolytic oscillations in the yeast extract support this hypothesis but also reveal several previously underestimated factors that may be essential for understanding the transition from prebiotic chemistry to biochemistry.

Being an evolutionarily ancient pathway, glycolysis is viewed as a living biochemical fossil that has retained features potentially relevant to prebiotic systems [21]. One of the first key observations in this study was that an oscillating extract was obtained only after a specific starvation stage (*Fig. 1*). This finding seems to indicate that the oscillatory regime is not merely a metabolism byproduct but is rather a special dynamic state achieved at a particular metabolic balance: namely, at the ATP : NADH ratio ensuring the required distance from the equilibrium (*Fig. 2*). Interestingly, it is NADH, rather than NAD^+^, that is critical for initiating the oscillation of the system. This fact can indicate that the oscillatory circuit is sensitive to the redox potential. By inhibiting certain enzymes in the upper part of glycolysis and acting as an alcohol dehydrogenase substrate, NADH may generate the negative feedback loop required for oscillations, as several models have previously proposed [22]. Therefore, we have experimentally demonstrated that the preliminary formation of a sufficient pool of reducing equivalents and energy-rich compounds within the system is a prerequisite for switching to the dissipative self-organization regime.

The robustness of the established oscillations to perturbations is no less important. The finding that the addition of most intermediates did not disrupt their rhythm (*Fig. 3B,C*) attests to the high degree of the system’s internal connectivity and buffering capacity. This feature may have been critically important for early metabolic protosystems encountering fluctuations in substrate concentrations in their environment. The ability to maintain an internal rhythm regardless of external fluctuations would have enhanced stability and survival.

Addition of pyruvate, and especially PEP, had the most dramatic effect (*Figs. 2A, 3*). The fact that these very metabolites inhibited oscillations shifts the emphasis in regulation from the classical central component, PFK, to the pyruvate kinase-catalyzed terminal reaction. Whereas PFK is often considered the major oscillator due to its complex allosteric regulation [22], pyruvate kinase, whose activity depends on PEP and is inhibited by ATP, may be a key sensor halting the system in response to product accumulation. This provides grounds for viewing the glycolytic oscillator not as a single-point generator but rather as a distributed network where multiple enzymes contribute to oscillation generation and quenching. The evolutionary antiquity of the lower part of glycolysis, up to the presence of only enolase and pyruvate kinase in some archaea [23], supports the hypothesis that primitive energy-producing cycles may have relied on the transformations of C_3_ compounds, and that the PEP/pyruvate pair could have played a key regulatory role in them.

Switching to the microvolumes in our experiments resulted in a qualitative leap: the lag phase disappeared (*Fig. 4*). This finding makes it necessary to reconsider the roles of diffusion and compartmentalization. In macrovolumes, the fact that it takes a certain time for self-sustaining oscillations to be established may be related not only to attaining the threshold metabolite concentrations, but also to the slow self-organization of enzyme complexes. Under cell-like conditions (micron-sized dimensions), the diffusion pathways are shortened while the local concentrations of enzymes with respect to one another increase, which may abruptly accelerate the formation of weak, transient enzyme–enzyme assemblies (metabolons) [24, 25, 26]. Therefore, spatial confinement, which is an inherent property of protocells, may *per se* have been a factor that not only concentrated the reactants, but was also involved in the promotion of complex dynamics and coordinated behavior in chemical networks.

The most intriguing finding, quite unexpected for us, was the impact of exogenous NAs on the oscillation kinetics. The nonspecificity of this effect (the identical impacts of RNA and DNA of different structures) makes us reject the hypothesis about a riboswitch-type or an aptamer-type interaction. Importantly, translation is impossible in our system, so the observed effect is direct. We are inclined to interpret it as a manifestation of nonspecific physicochemical interactions. However, their nature is still to be elucidated in simpler models using individual purified components of the system. This mechanism could provide fast, non-genomic feedback between the gene expression landscape and fine-tuning of the existing metabolic networks.

Therefore, our study shows that even a relatively simple reconstituted metabolic system can exhibit complex dynamics depending on the ratio between metabolite concentrations, the spatial organization of the components, and the biophysical context. Based on our findings, we propose a more detailed scenario of early biochemical evolution comprising several potentially sequential stages: (1) the formation of primitive catabolic cycles based on C_3_ compounds, regulated by feedback inhibition; (2) the stabilization and synchronization of these cycles upon microcompartmentalization, which contributed to the emergence of stable oscillations; and (3) the coevolution with emerging polymers (RNA), which had initially exerted a nonspecific physical impact that evolved into specific regulation and information storage only later on.

## CONCLUSIONS

In our study, we reconstituted the oscillating glycolytic pathway in a cell-free system derived from the *S. cerevisiae* yeast. The concentrations of metabolites (ATP and NADH) were found to be the key factors responsible for the emergence and maintenance of oscillations. Periodic changes were observed not only for NADH but also for glucose, hexose-6-phosphates, glyceraldehyde-3-phosphate, 3-phosphoglycerate, and phosphoenolpyruvate (PEP). PEP was found to play a key regulatory role. The role played by compartmentalization in the establishment and maintenance of the oscillatory dynamics of the processes was demonstrated using a microfluidic chip. We established, for the first time, that glycolysis is regulated by RNA and DNA; this effect is dependent on neither the secondary structure nor the nucleotide sequence of nucleic acids.

## Abbreviations

HPLC/MS: high-performance liquid chromatography/mass spectrometry
NAs: nucleic acids
PK: pyruvate kinase
PEP: phosphoenolpyruvate
PFK: phosphofructokinase
PDMS: polydimethylsiloxane
POPC: (1-palmitoyl-2-oleoyl-sn-glycero-3-phosphocholine)
